# Multiseptate Gallbladder in an Asymptomatic Child

**DOI:** 10.1155/2011/470658

**Published:** 2011-09-15

**Authors:** Dylan Wanaguru, Ashish Jiwane, Andrew S. Day, Susan Adams

**Affiliations:** ^1^Department of Paediatric Surgery, Sydney Children's Hospital, University of New South Wales, High Street, Randwick, Sydney, NSW 2031, Australia; ^2^Department of Gastroenterology, University of New South Wales, High Street, Randwick, NSW 2031, Australia; ^3^Sydney Children's Hospital and School of Women's and Children's Health, University of New South Wales, High Street, Randwick, NSW 2031, Australia

## Abstract

A one-year-old child being investigated for urinary tract infection was diagnosed with a multiseptate gallbladder. The patient remains asymptomatic, and investigations demonstrate no associated anomalies. Forty-three cases, including 13 cases in children were identified in the literature. Their presentation and management were reviewed.

## 1. Introduction

Multiseptate gallbladder (MSG) is a rare congenital anomaly with less than 50 cases described in the English literature. Of these, 13 are in paediatric patients [1–9]. We report a case of MSG in a one-year-old child. We review the data from the published literature to consider the most appropriate management of symptomatic and asymptomatic children, including whether or not cholecystectomy is indicated.

## 2. Case Report

**Figure 1:**
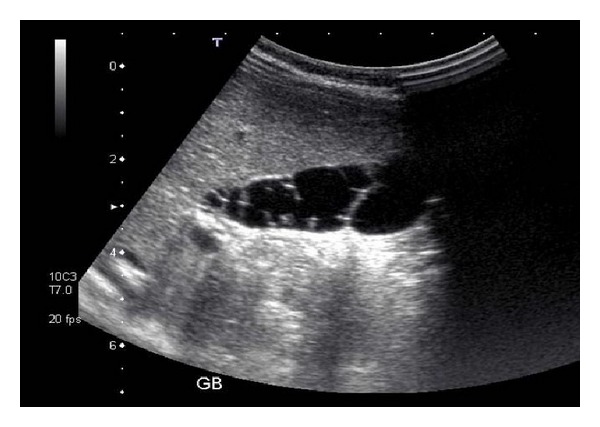
Ultrasound image demonstrating multiple fine septations within the gallbladder.

A nine-month-old, previously well, female infant presented with an acute episode of vomiting and was diagnosed with an *Escherichia coli *urinary tract infection (UTI). Renal tract ultrasound was normal, but the gallbladder (GB) was incidentally noted to have “multiple thin smooth septa, giving a honeycomb appearance,” consistent with MSG (Figure 1). No other biliary tract abnormality was noted. The UTI was treated, and the child subsequently remained asymptomatic.

Repeat ultrasound six months later showed the images were unaltered. Liver function tests were normal apart from a raised alkaline phosphatase 1632 U/L (age appropriate normal range 80–450). Magnetic resonance cholangiopancreaticography (MRCP) confirmed the diagnosis of MSG, and excluded intra- and extrahepatic biliary and pancreatic anomalies. Nuclear medicine HIDA (hepatobiliary imino-diacetic acid) scan revealed no evidence of obstruction to bile flow at any level of the biliary tree.

## 3. Discussion

Multiseptate gallbladder was first described in 1963 by Simon and Tandon [10]. It is characterised by multiple thin septations within the gallbladder lumen, giving a honeycomb-like appearance. Simon and Tandon [10] proposed that this was due to incomplete vacuolisation of the developing gallbladder bud. Bhagavan et al. [11] have suggested that MSG may be a result of the solid embryonic GB growing faster than its bed and investing peritoneum, causing aberrant bends and kinks. The same authors also postulate that a variation in the wrinkling, lobulation, and clefting of the gallbladder (seen in cat and guinea pig embryos) may result in multiseptation [11].

Including the current case, 44 cases are described in the English literature, with a male-to-female ratio of almost 1 : 2. Overall the mean age at diagnosis of these individuals was 28.6 years (range from 15 days to 70 years). Thirty of these cases were in adults [10–36]. Thirteen have been reported in children: eight being female (Table 1) [1–9]. Most children were diagnosed in mid-to-late childhood (mean age 9.4 years) although one was detected at 15 days of age.

Biliary symptoms such as right upper quadrant pain, nausea and vomiting are the most common complaints in this condition, with 31 of the 44 cases presenting in this manner. Only three of the 44 cases were associated with cholelithiasis [8, 12, 13], and one was associated with acute acalculous cholecystitis [14], but none of these were in children. The presence of an associated biliary tract abnormality is an important consideration in the assessment of MSG. One case in a 46-year-old woman was associated with anomalous pancreaticobiliary ductal union [15]. The three reported cases found to have associated choledochal cysts were all in children (23% of paediatric cases). These children presented with jaundice [2, 9] and a combination of fever, nausea, and abdominal discomfort [6]. Seven of the remaining 10 children presented with biliary symptoms [1, 3, 5, 7, 8]. The other three children (including the current case) were asymptomatic with no biliary tract anomaly [4, 5]. The incidence of asymptomatic MSG in the community is unknown, so it is not possible to comment on the likelihood of symptoms developing in these children. 

There is no reported association between uncomplicated MSG and malignancy; however, there is a known link between biliary tract anomalies and cholangiocarcinoma. The incidence of malignancy in choledochal cyst is reported between 10% and 30%, and anomalous arrangement of the pancreaticobiliary duct is considered to be a high-risk factor for biliary tract malignancy [16, 17]. Consequently, four of the reported cases of MSG (all adults) with such associated abnormalities have an increased long-term risk of malignancy.

The majority of reported cases were diagnosed on ultrasound. Differential diagnosis includes desquamated gallbladder mucosa, polypoid cholesterolosis, hydatid cyst and acute hepatitis [18, 19]. Kocakoc et al. [7] first reported the use of MRCP to noninvasively define biliary and pancreatic pathology in MSG. MRCP is useful in confirming the diagnosis and delineating any associated biliary tree pathology and eliminates the potential complications associated with endoscopic cholangiopancreaticography (ERCP). 

Saimura et al. [19] conducted biliary manometry and scintigraphy on a 30-year-old man with epigastric pain and MSG. Impairment of bile flow into and out of the gallbladder was demonstrated, supporting a biliary origin of the patient's pain. In the same study, Saimura and colleagues went on to reproduce typical biliary colic in the patient by injection of Cerulein to stimulate gallbladder contraction. 

In symptomatic patients diagnosed with MSG, cholecystectomy provides relief of symptoms [21]. The three children with associated choledochal cyst were successfully treated with excision of the extrahepatic biliary tree combined with hepatojejunostomy or choledochoduodenostomy. In the 12 reported cases of asymptomatic and uncomplicated MSG, including the three paediatric cases, management has been nonoperative with regular followup.

## 4. Conclusion

MSG is a rare biliary anomaly that may be diagnosed in the first decades of life. Associated biliary tract anomalies should be excluded, particularly when the diagnosis is made in childhood. Cholelithiasis is rarely associated with MSG, and has never been reported in a child. Investigation with ultrasound, MRCP, and HIDA scan is recommended. In the absence of symptoms attributable to the MSG, or an associated biliary tract anomaly, nonoperative management in children and regular followup is reasonable. Symptomatic uncomplicated MSG is successfully treated with cholecystectomy.

## Figures and Tables

**Figure 2:**
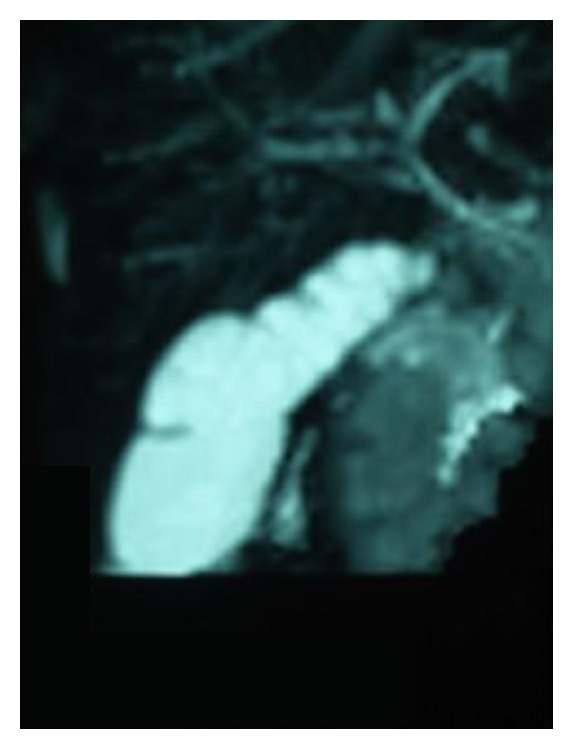
Magnetic Resonance cholangiopancreaticography (MRCP) image confirming ultrasound findings of multiple septae within the gallbladder.

**Table 1:** Published reports of multiseptate gallbladder in children and adolescents (aged less than 16 years).

Author (reference)	Year	Age	Sex	Biliary symptoms	Associated anomalies	Treatment
Haslam et al. [1]	1966	15	F	Yes	Nil	Cholecystectomy
Pery et al. [2]	1985	8	F	Yes	Choledochal cyst	Cholecystectomy and choledochoduodenostomy
Fremond et al. [3]	1989	13	F	Yes	Nil	Cholecystectomy
Adear and Barki [4]	1990	12	F	No	Nil	Nil
Strauss et al. [5]	1993	3	M	No	Nil	Not detailed
Strauss et al. [5]	1993	9	F	Yes	Nil	Not detailed
Strauss et al. [5]	1993	16	M	Yes	Nil	Not detailed
Tan et al. [6]	1993	14	F	Yes	Choledochal cyst	Cholecystectomy and hepatojejunostomy
Kocakoc et al. [7]	2003	9	M	Yes	Nil	Cholecystectomy
Erdogmus et al. [8]	2004	10	F	Yes	Nil	Cholecystectomy
Erdogmus et al. [8]	2004	12	M	Yes	Nil	Cholecystectomy
Bahadir et al. [9]	2006	15 days	M	Yes	Ectopic pancreas associated with choledochal cyst	Total excision of cyst with Roux-en-Y anastomosis
Present Case	2008	1	F	No	Nil	Monitor with ultrasound
